# miR-671-5p as a diagnostic biomarker and therapeutic target in periodontitis via THBS1 regulation

**DOI:** 10.1186/s41065-025-00546-w

**Published:** 2025-09-25

**Authors:** Shan Huang, Wei Cheng, Jianlan Deng

**Affiliations:** 1 Oral Medicine Center, Hengqin Hospital, The First Affiliated Hospital of Guangzhou Medical University , Guangzhou, Guangdong 519000 China; 2 Department of Stomatology, Sinopharm Dongfeng Maojian Hospital, Shiyan, Hubei 442000 China; 3 Department of Stomatology, Nanjing Hospital of C.M, Nanjing Hospital of Chinese Medicine Affiliated to Nanjing University of Chinese Medicine , No. 157, Daming Road, Qinhuai District, Nanjing, 210022 China

**Keywords:** Periodontitis, miR-671-5p, THBS1, Inflammation, Diagnostic biomarker

## Abstract

**Background:**

Early diagnosis and therapeutic targeting of periodontitis remain challenging. This study aimed to investigate the clinical relevance and mechanistic role of miR-671-5p in pathogenesis of periodontitis.

**Methods:**

Clinical data and gingival crevicular fluid (GCF) samples were collected from 78 periodontitis patients and 79 healthy controls. miR-671-5p expression in GCF was quantified via quantitative reverse transcription polymerase chain reaction (qRT-PCR), and the diagnostic efficacy was evaluated by receiver operating characteristic (ROC) analysis. To model inflammation, lipopolysaccharide (LPS)-stimulated human periodontal ligament fibroblasts (hPDLFs) were employed. Subsequently, miR-671-5p mimics or inhibitors were transfected to assess effects on cell viability assessed by CCK-8, cellular migration measured via Transwell assays, and cytokine secretion analyzed using enzyme-linked immunosorbent assay (ELISA). THBS1 targeting by miR-671-5p was validated via dual-luciferase assays.

**Results:**

miR-671-5p expression was significantly reduced in GCF of periodontitis patients, and a strongly correlation was observed with clinical severity indices. ROC analysis revealed high diagnostic accuracy. Furthermore, miR-671-5p levels exhibited an inverse correlation with TNF-α, IL-6, and IL-1β. In LPS-treated hPDLFs, miR-671-5p was associated with a dose-dependent reduction in cell viability and migration, as well as an increase in the production of inflammatory cytokines. miR-671-5p was found to directly target THBS1 mRNA, inhibiting its expression. miR-671-5p overexpression reversed LPS-induced functional impairment and inflammation; these beneficial effects were partially counteracted by THBS1 upregulation.

**Conclusions:**

miR-671-5p holds promise as a potential diagnostic biomarker and therapeutic target in periodontitis by regulating THBS1-mediated inflammatory responses and dysfunction of hPDLFs.

**Supplementary Information:**

The online version contains supplementary material available at 10.1186/s41065-025-00546-w.

## Background

Delayed diagnosis remains a critical barrier in periodontitis management, often leading to irreversible tissue destruction before clinical intervention. Consider a 45-year-old patient presenting with gingival bleeding; current diagnostic tools fail to differentiate between early active disease and gingivitis, raising the risk of progression to bone loss. A rapid chairside test capable of detecting pathogenic miRNA signatures in gingival crevicular fluid (GCF) holds the potential to revolutionize this situation by facilitating pre-destructive intervention. Periodontitis, initiated by plaque biofilms, progressively destroys periodontal tissues through alveolar bone resorption and periodontal ligament degradation [1]. Affecting over 50% of adults worldwide and identified as the leading cause of tooth loss [2], its silent progression underscores urgent needs for early-diagnosis tools. Conventional therapies, such as mechanical debridement, provide only temporary control of inflammation with recurrence rates ranging from 30 to 50%, and they fall short in regenerating lost tissues [3, 4]. Consequently, the identifying of biomarkers for point-of-care detection, as well as novel therapeutic targets, emerges as a pressing clinical imperative.

Human periodontal ligament fibroblasts (hPDLFs) serve as the primary functional cells within periodontal tissues, playing a pivotal role in maintaining extracellular matrix homeostasis [5, 6]. However, during the progression of periodontitis, pathogen-associated molecular patterns, such as lipopolysaccharide (LPS), can induce a phenotypic transformation in hPDLFs. This transformation triggers an augmented secretion of matrix metalloproteinases (MMPs) and pro-inflammatory cytokines like IL-6 and TNF-α [7]. Concurrent activation of the nuclear factor kappa B (NF-κB) signaling pathway occurs. These changes directly contribute to collagen degradation and bone resorption [8]. Therefore, elucidating the molecular network that underpins hPDLFs dysfunction is essential. Such insights are critical for development of strategies aimed at mitigating tissue destruction and facilitating repair.

MicroRNAs (miRNAs) regulate gene expression post-transcriptionally. They exert precise control over pathological processes, including inflammation, apoptosis, and tissue repair [9]. Growing evidence confirms that aberrant miRNA expression closely links to the onset and progression of periodontitis. For instance, miR-21 alleviates periodontal tissue inflammation by inhibiting pro-inflammatory cytokine release and extracellular matrix degradation [10]. Conversely, miR-204-5p regulates hPDLFs function by targeting Dicckopf-1 (DDK1), thereby influencing periodontitis development [11]. Recently, miR-671-5p has garnered attention for its regulatory roles in inflammation and fibrosis. Research demonstrates its protective effects in diverse models. Within ischemic stroke models, it mitigates neuroinflammation by modulating the NF-κB pathway [12]. Furthermore, it suppresses the activation of fibroblasts in myocardial fibrosis through the targeting FGFR2 [13]. Notably, a miRNA array study has identified dysregulated l miR-671-5p expression in periodontitis patients [14]. However, its specific role and underlying mechanisms within the context of periodontitis remain to be fully elucidated. Given this functional profile, the potential significance of miR-671-5p in the pathogenesis of periodontitis necessitates urgent investigation.

This study presents the first systematic exploration of miR-671-5p in periodontitis. We evaluated its diagnostic potential and elucidated its regulatory mechanism on hPDLFs function. First, we compared miR-671-5p expression levels in GCF between healthy controls and periodontitis patients to assess clinical relevance. Subsequently, using an LPS-induced hhPDLFs inflammation model, we employed gain-and loss-of-function experiments. This research offers novel avenues for early diagnosis and targeted therapy of periodontitis.

## Methods

### Study subjects

A total of 78 periodontitis patients treated at Nanjing Hospital of C.M. between 2020 and 2023 were enrolled, along with 79 age-matched healthy controls with normal gingival tissues during the same period. Inclusion criteria for patients were: (1) diagnosis meeting established periodontitis criteria [15]; (2) age 18 years or older with initial diagnosis; (3) no oral disease treatment within the preceding 6 months; (4) complete clinical and follow-up records; (5) voluntary participation. Exclusion criteria applied to all participants were: (1) females during menstruation, lactation, or pregnancy; (2) poor treatment adherence; (3) concurrent neoplastic disease; (4) refusal of periodontal tissue sampling; (5) concurrent oral diseases such as periapical lesions or aggressive periodontitis. The Institutional Review Board of Nanjing Hospital of C.M. approved this study.

### Cell culture and transfection

hPDLFs (human periodontal ligament fibroblasts, purchased from Shanghai Nuochen Biotechnology Co., China) were cultured in Dulbecco’s Modified Eagle Medium (DMEM) supplemented with 10% fetal bovine serum. The cultures were maintained at 37 °C in a humidified atmosphere containing 5% CO₂. At the logarithmic growth phase, with a confluence of approximately 80%, the cells were exposed to LPS at concentrations of 0, 1, 2, or 3 µg/mL for a duration of 24 h to ascertain the optimal LPS concentration for subsequent experiments. miR-671-5p mimic (miR-671-5p), negative control (miR-NC), THBS1 overexpression plasmid (OE-THBS1), and control plasmid (OE-NC) were obtained from Guangzhou Ribobio Co. For the transfection process, Lipofectamine 3000 (Invitrogen, USA) was utilized to facilitate the introduction of these constructs into hPDLFs, allowing for a 48-hour at 37 °C. Control groups were established, consisting of non-transfected cells not subjected to LPS treatment and non-transfected cells that received LPS exposure.

### Quantitative real-time PCR (qRT-PCR)

GCF samples from subjects and hPDLFs from each group were collected for total RNA utilizing Trizol reagent. Subsequently, RNA isolation was performed employing the mirPremier microRNA Isolation Kit (BioRad, USA), followed by reverse transcription to synthesize cDNA using the SuperScript RT Kit (Yuanmu Biotechnology Co., Ltd., China). The resultant cDNA served as the template for PCR amplification, conducted with SYBR Green PCR master Mix (TaKaRa Bio Inc., China) in strict adherence to the manufacturer’s protocols. The expression levels of miR-671-5p and THBS1 mRNA were normalized against U6 and β-actin, respectively. Relative expression levels were calculated using the 2^-ΔΔCt^ method.

### Cell migration assay

hPDLFs in the logarithmic growth phase were resuspended in serum-free DMEM and subsequently seeded into the upper chambers of Transwell plates. The lower chambers were filled with DMEM containing 10% fetal bovine serum. After a 24-hour incubation period, non-migrated cells were removed. The migrated cells were then fixed with 4% paraformaldehyde for 10 min, followed by staining with 0.5% crystal violet. Excess stain was gently washed with tap water, and the migrated cells were counted using an inverted microscope.

### Dual-luciferase reporter assay

Amplified sequences were cloned into the pmirGLO dual-luciferase vector (Promega, USA) to generate wild-type (WT-THBS1, containing the miR-671-5p binding site) and mutant (MT-THBS1, with a mutated binding site) reporter constructs for the THBS1 3’UTR. These WT or MT vectors were co-transfected with miR-671-5p mimic or control into hPDLFs (ATCC, USA) using Lipofectamine 3000 as the transfection reagent. Luciferase activity was assessed 48 h after transfection using a dual-luciferase reporter assay system (Solarbio, China).

### Cell viability assay

Cell viability was evaluated using a CCK-8 kit (Thermo Fisher Scientific, USA). Cells were plated at a density of 1 × 10³ cells per well in 96-well plates. After a 72-h culture incubation period, 10 µL of CCK-8 solution was introduced to each well. Following an additional 2-hourincubation at 37 °C, he optical density at 450 nm (OD_450_) was measured using a SpectraMax M5 plate reader (Molecular Devices, USA).

### Inflammatory cytokine measurement

The levels of interleukin-6 (IL-6), interleukin-1β (IL-1β), and tumor necrosis factor-alpha (TNF-α) were quantified in GCF samples a obtained from patients, as well as in the supernatants of cell cultures. The supernatants were collected after centrifugation at 12, 000 rpm for 15 min to ensure optimal separation of the cellular debris. Quantitative measurements were carried out utilizing specific enzyme-linked immunosorbent assay (ELISA) kits sourced from Solarbio (Beijing, China), adhering strictly to the protocols prescribed by the manufacturer.

### NF-κB inhibition assay

hPDLFs were subjected to pre-treated with 10 µM BAY11-7082 (MedChemExpress, USA) or an equivalent volume of DMSO as a solvent control for a duration of 2 h prior to stimulation with LPS and/or the overexpression of THBS1. Following the treatments, cells were harvested 24 h later for subsequent analyses of protein and cytokine analysis. To extract total protein from the treated hPDLFs, RIPA lysis buffer was employed, supplemented with protease and phosphatase inhibitors to ensure protein integrity. Protein concentrations were determined via bicinchoninic acid (BCA) assay (Thermo Fisher Scientific, USA) according to the manufacturer’s protocol. Proteins were separated by 10% SDS-polyacrylamide gel electrophoresis (SDS-PAGE) and transferred to polyvinylidene difluoride (PVDF) membranes using a semi-dry transfer system. Membranes were blocked with 5% (w/v) non-fat milk in TBST for 1 h at room temperature. Primary antibodies against phosphorylated p65 (p-p65; 1:1,000), total p65 (1:1,000), and β-actin (Abcam, 1:5,000) were diluted in TBST and applied with membranes overnight incubation at 4 °C. After TBST washes, HRP-conjugated secondary antibodies (1:10,000) were applied for 1 h at room temperature. Protein bands were visualized using an ECL substrate kit (Bio-Rad, USA) and imaged with a ChemiDoc system (Bio-Rad). The intensities of the bands were quantified using ImageJ software (NIH, USA), employingβ-actin as the internal loading control.

### Statistical analysis

Data analysis was conducted utilizing SPSS Statistics software, version 21.0 (IBM, USA). Continuous variables are presented as mean ± standard deviation (x ± s). The comparison of differences between the two groups was carried out using the independent samples t-test, while the one-way analysis of variance (ANOVA) was employed for assessments among multiple groups. Pearson correlation analysis was used to assess relationships between inflammatory cytokine levels and miR-671-5p expression in periodontitis patients. Statistical significance was defined as *P* < 0.05.

## Results

### miR-671-5p expression is downregulated in the GCF of periodontitis patients and demonstrates diagnostic value

This study enrolled 99 healthy control subjects and 98 periodontitis patients. Baseline characteristics, including age, BMI, sex, and smoking or alcohol consumption habits, showed no significant differences between the two groups (Table 1). Clinical examinations verified that healthy controls satisfied the criteria for periodontal health: each individual displayed a probing depth (PD) of less than 3 mm, a clinical attachment loss (CAL) of 0 mm, and a bleeding on probing (BOP) rate of less than 10%. Conversely, periodontitis patients demonstrated markedly elevated PD, CAL, and BOP rates (*P* < 0.001).

**Table 1:** Clinical data of the study subjects

Clinical Information	Healthy control	Periodontitis	*P* value
	(*n* = 79)	(*n* = 78)	
Age (years)	42.68 ± 9.86	43.62 ± 9.33	0.544
BMI (kg/m^2^)	23.47 ± 1.45	24.75 ± 1.78	0.282
Gender (female/male)	42/37	39/39	0.692
Dietary favor (light/heavy)	40/39	44/34	0.468
Smoking history (yes/no)	41/38	45/33	0.466
Drinking history (yes/no)	38/41	43/35	0.378
PD (mm)	1.98 ± 0.60	4.90 ± 1.12	< 0.001
CAL (mm)	0	3.96 ± 1.52	< 0.001
BOP (%)	7.23 ± 1.64	40.41 ± 14.56	< 0.001

BMI: Body Mass Index

PD: Periodontal Pocket Depth

CAL: Clinical Attachment Loss

BOP: Bleeding on Probing

**Fig. 1:**
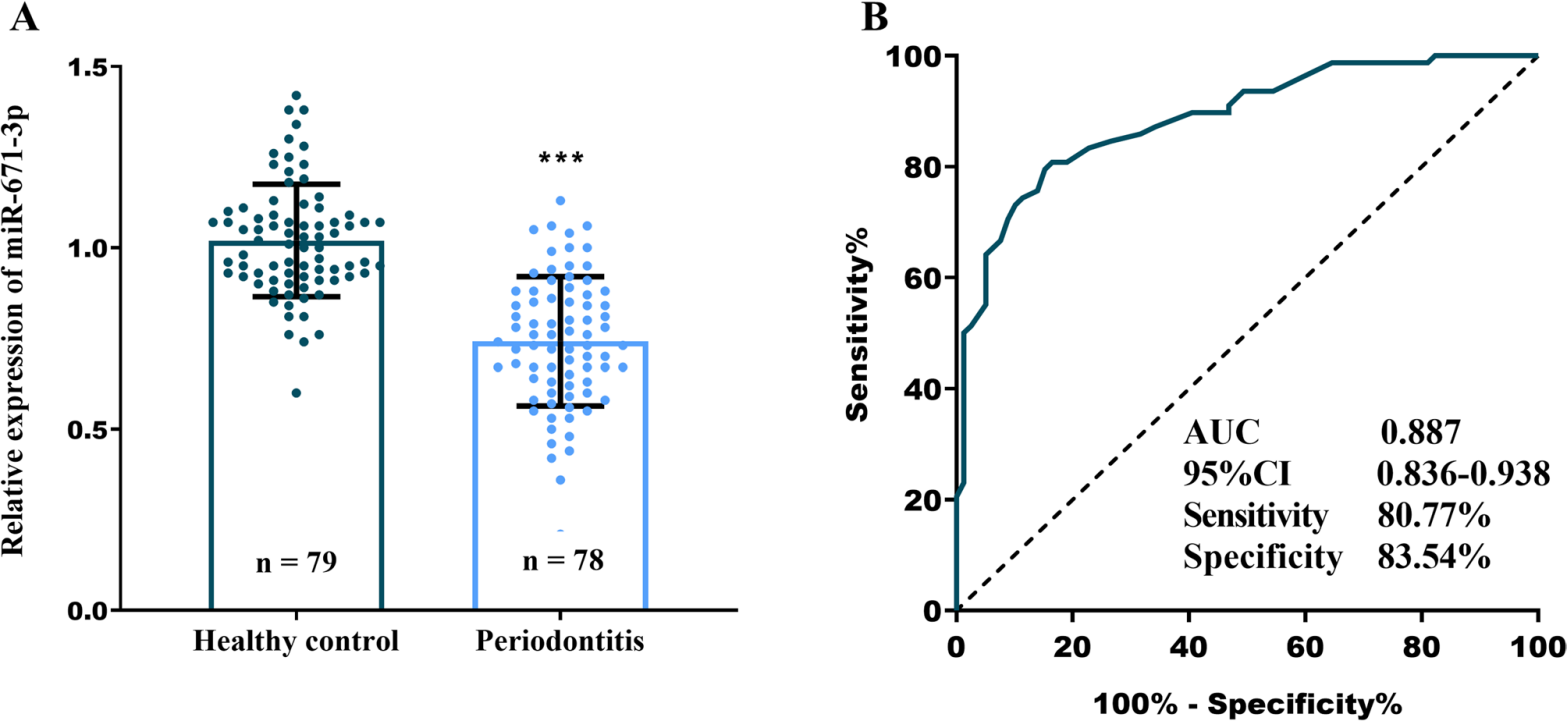
GCF miR-671-5p expression and diagnostic utility in periodontitis. (**A**) miR-671-5p levels in healthy vs. periodontitis GCF. (**B**) ROC curve assessing miR-671-5p diagnostic accuracy for periodontitis. *** *P* < 0.001 vs. healthy

Analysis revealed that the expression levels of miR-671-5p in GCF were significantly diminished in periodontitis patients when compared to healthy controls (Fig. 1A, *P* < 0.001). ROC curve analysis demonstrated that miR-671-5p effectively distinguished healthy controls from periodontitis patients, achieving an area under the curve (AUC) of 0.887 (95% CI: 0.836–0.938; Fig. 1B), which underscores its excellent diagnostic performance. Normality testing (Shapiro-Wilk) confirmed miR-671-5p expression data were normally distributed (*P* > 0.05). Periodontitis patients were stratified into high and low miR-671-5p expression groups based on the mean expression level. The low expression group exhibited significantly higher proportions of cases with PD ≥ 5 mm (74%), CAL ≥ 4 mm (76%), and BOP ≥ 40% (69%) in comparison to the high expression group (*P* < 0.05; Table 2).

**Table 2:** Association of miR-671-3p with periodontitis patients’ clinicopathological features

Variant	Cases (*n* = 78)	miR-671-3p expression		*P* value
		Low (*n* = 40)	High (*n* = 38)	
PD (mm)				< 0.001
< 5	40	12 (30%)	28 (70%)	
≥ 5	38	28 (74%)	10 (26%)	
CAL (mm)				< 0.001
< 4	40	11 (27%)	29 (73%)	
≥ 4	38	29 (76%)	9 (24%)	
BOP (%)				0.001
< 40	36	11 (31%)	25 (69%)	
≥ 40	42	29 (69%)	13 (31%)	

PD: Periodontal Pocket Depth

CAL: Clinical Attachment Loss

BOP: Bleeding on Probing

**Fig. 2:**
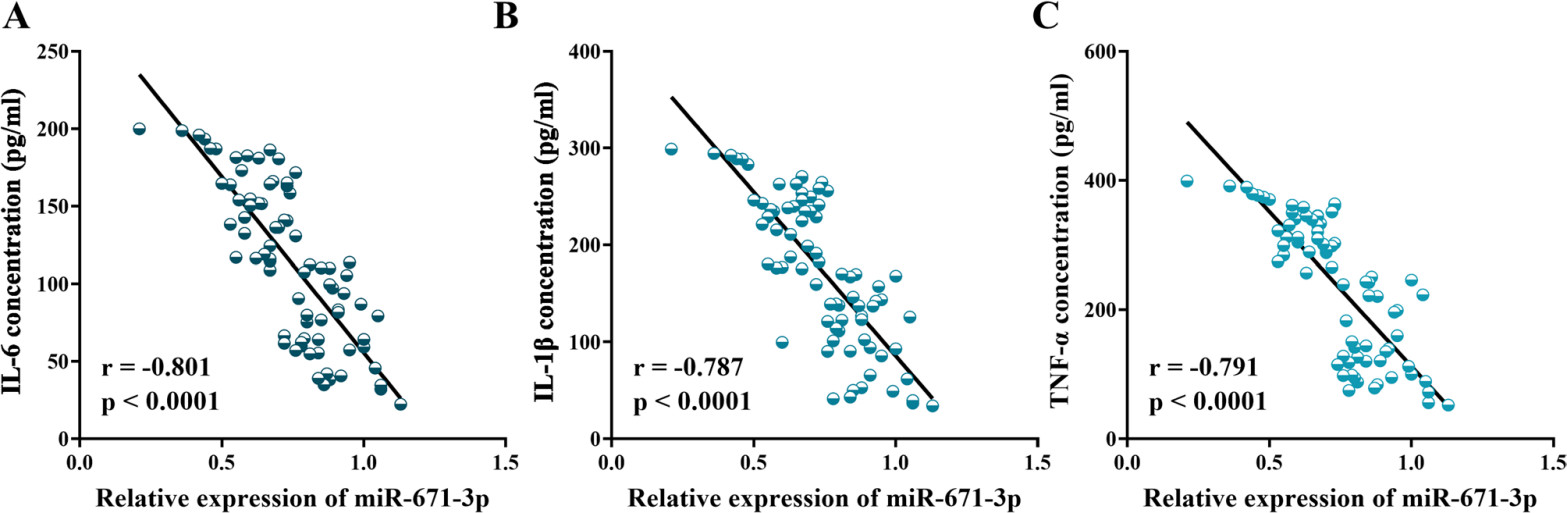
Inverse correlation of GCF miR-671-5p with inflammatory cytokines. The scatter plots illustrate the negative correlations observed between GCF miR-671-5p and the concentrations of (**A**) IL-6, (**B**) IL-1β, (**C**) TNF-α

Furthermore, correlation analysis demonstrated a significant negative correlation between miR-671-5p expression levels and GCF concentrations of IL-6 (*r* = -0.801, *P* < 0.001; Fig. 2A), IL-1β (*r* = -0.787, *P* < 0.001; Fig. 2B), and TNF-α (*r* = -0.791, *P* < 0.001; Fig. 2C).

### The LPS-induced inflammatory microenvironment suppresses miR-671-5p expression and impairs hPDLFs function

**Fig. 3:**
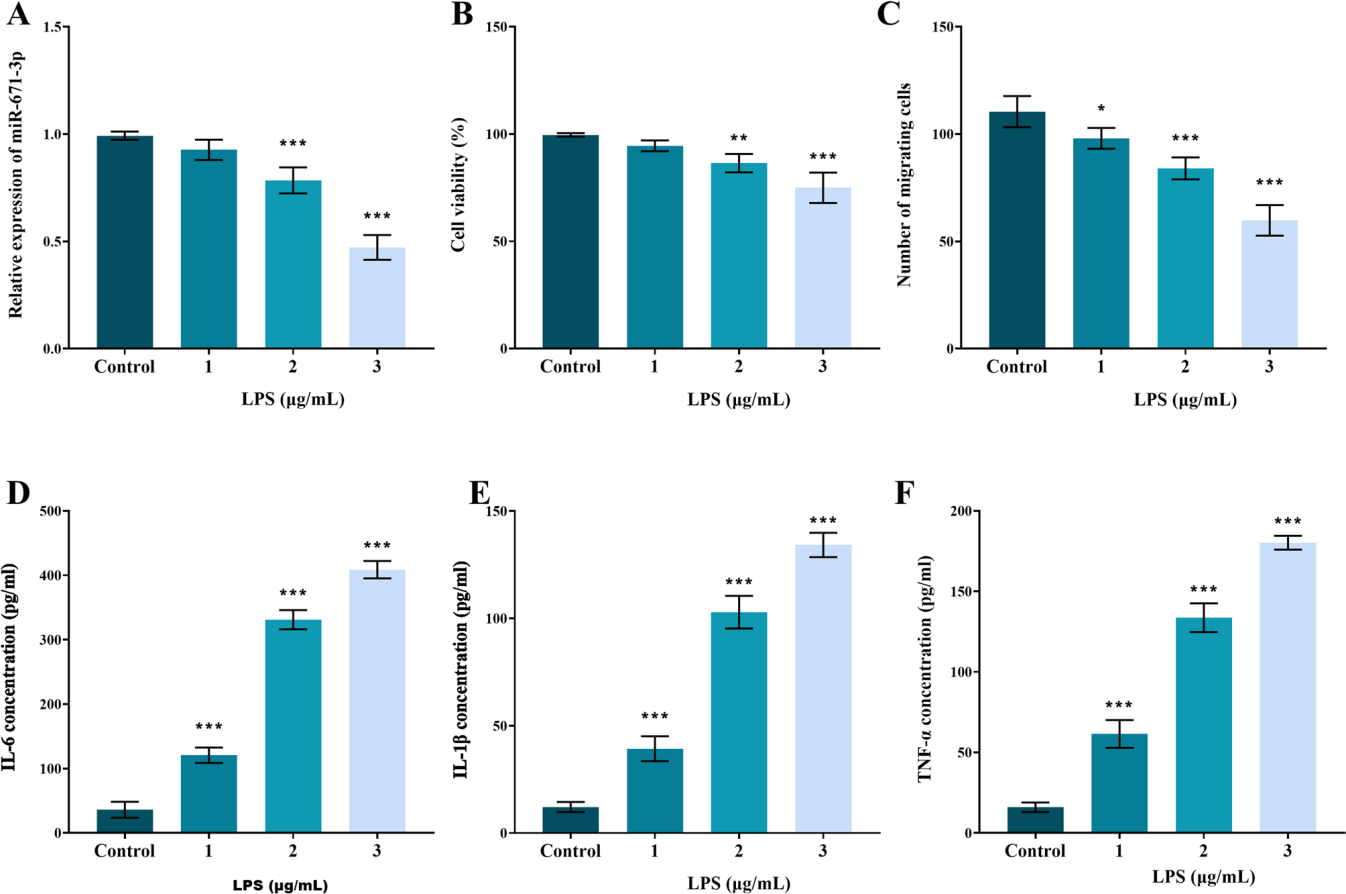
LPS dose-effects on miR-671-5p and hPDLFs function. (**A**) Dose-dependent miR-671-5p downregulation in hPDLFs by LPS. LPS progressively reduces (**B**) viability and (**C**) migration, while increasing supernatant (**D**) IL-6, (**E**) IL-1β, (**F**) TNF-α. *P* < 0.01, ** *P* < 0.001 vs. 0 µg/mL LPS (Control)

In vitro experiments employed varying concentrations of LPS ranging from 0 to 3 µg/mL to stimulate hPDLFs and model inflammation responses. Data revealed that miR-671-5p expression in hPDLFs decreased in a concentration-dependent pattern as LPS levels increased (Fig. 3A, *P* < 0.05). Cell viability (Fig. 3B) and migration capacity (Fig. 3C) also progressively declined with higher LPS concentrations (*P* < 0.05). Concurrently, levels of IL-6, IL-1β, and TNF-α in the cell culture supernatant increased significantly with rising LPS concentration (Figs. 3D-F, *P* < 0.001). Based on the dose-response profiles in Fig. 3, a concentration of 3 µg/mL LPS was selected for subsequent functional assays, thus achieving a robust induction of inflammation while maintaining cellular viability at approximately 75% of control levels.

### miR-671-5p directly targets THBS1 and regulates its expression

**Fig. 4:**
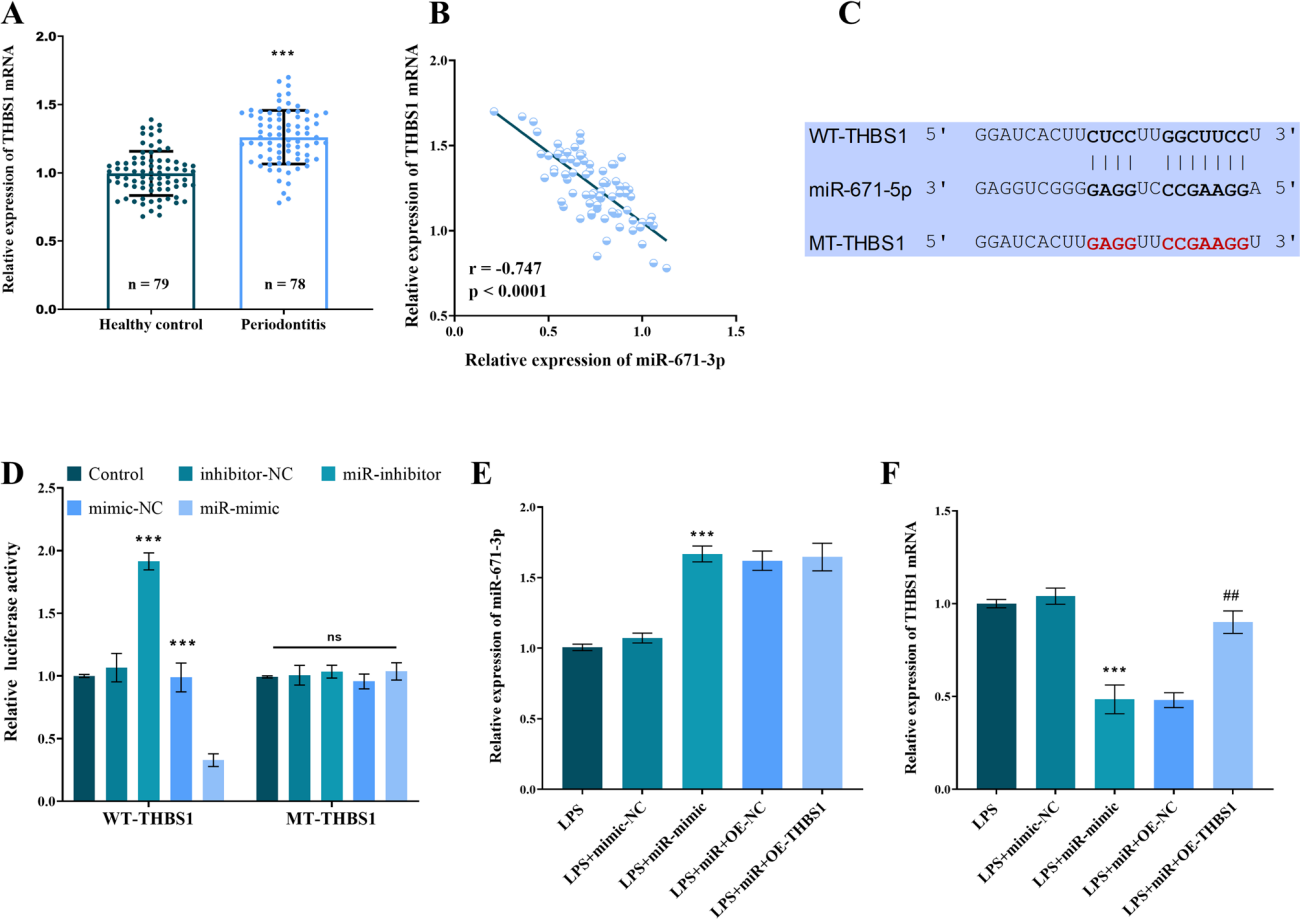
miR-671-5p directly targets THBS1. (**A**) Elevated GCF THBS1 in periodontitis vs. healthy. (**B**) Negative GCF THBS1/miR-671-5p correlation. (**C**) Predicted miR-671-5p binding site in THBS1 3’UTR. (**D**) Direct targeting confirmed by dual-luciferase assay. (**E**) miR-671-5p mimic increases expression in LPS-hPDLFs. (**F**) Mimic reduces THBS1; reversed by THBS1 overexpression. *** *P* < 0.001 vs. healthy/Control (Untreated hPDLFs)/LPS; ## *P* < 0.001 vs. LPS + miR-mimic. mimic-NC: Negative control for miR-671-5p mimic; miR: miR-671-5p mimic; OE-NC: Negative control for THBS1 overexpression; OE-THBS1: THBS1 overexpression

Analysis of clinical samples revealed that THBS1 levels in GCF were significantly higher in periodontitis patients than in healthy controls (Fig. 4A, *P* < 0.001). Furthermore, THBS1 levels showed a significant negative correlation with miR-671-5p expression (r = -0.747, *P* < 0.001; Fig. 4B). Bioinformatics predictions, alongside dual-luciferase reporter assays, confirmed that miR-671-5p directly interacts with the 3’ untranslated region (3’UTR) of THBS1 mRNA, which in turn negatively modulates luciferase activity (Figs. 4C-D, *P* < 0.01). Transfection with a miR-671-5p mimic significantly increased miR-671-5p expression in LPS-treated hPDLFs (Fig. 4E, *P* < 0.001) and suppressed THBS1 levels. Overexpression of THBS1 was able to reverse the inhibitory effect of the miR-671-5p mimic on THBS1 expression (Fig. 4F, *P* < 0.05). To further validate the regulatory effect of miR-671-5p inhibition, non-LPS-treated hPDLFs were transfected with a miR-671-5p inhibitor using Lipofectamine 3000. An assessment of THBS1 mRNA levels 48 h post-transfection revealed that the inhibitor significantly promoted THBS1 expression (*P* < 0.01 vs. control; Supplementary Figure S1), f thereby reaffirming the miR-671-5p-mediated regulation of THBS1.

### miR-671-5p improves cell function and attenuates inflammation by inhibiting THBS1

**Fig. 5:**
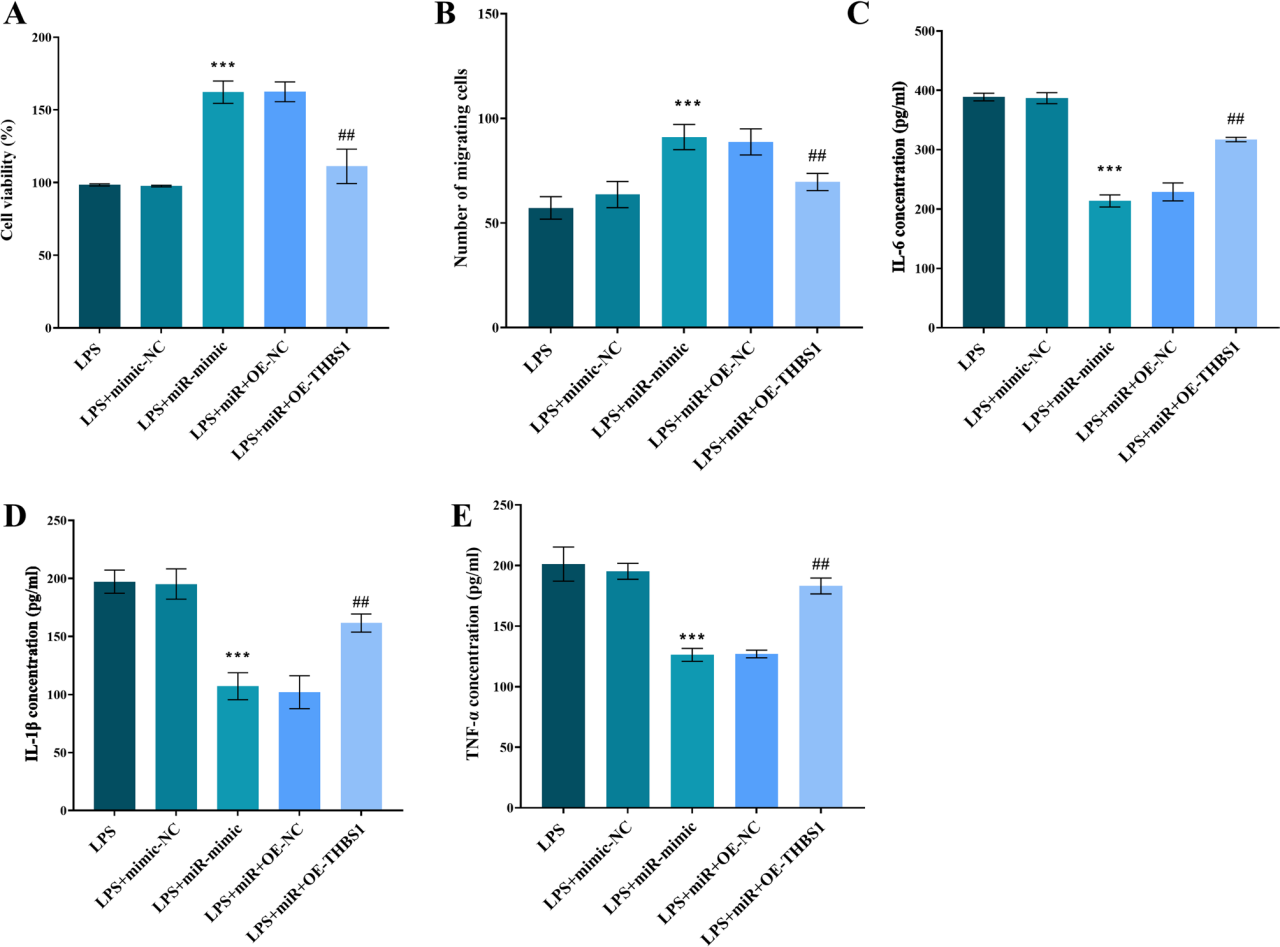
miR-671-5p overexpression rescues LPS-impaired hPDLF function; reversed by THBS1. miR-671-5p mimic in LPS-hPDLFs: enhances (**A**) viability and (**B**) migration; suppresses (**C**) TNF-α, (**D**) IL-6, (**E**) IL-1β secretion. THBS1 overexpression attenuates these effects. *** *P* < 0.001 vs. LPS; ## *P* < 0.001 vs. LPS + miR-mimic. mimic-NC: Negative control for miR-671-5p mimic; miR: miR-671-5p mimic; OE-NC: Negative control for THBS1 overexpression; OE-THBS1: THBS1 overexpression

Overexpression of miR-671-5p significantly enhanced the viability (Fig. 5A) and migration capacity (Fig. 5B) of LPS-treated hPDLFs. It also suppressed the secretion of TNF-α, IL-6, and IL-1β (Figs. 5C-E, *P* < 0.01). However, overexpression of THBS1 partially mitigated these protective effects associated with miR-671-5p. This r attenuation was evidenced by diminished cell viability, impaired migration capacity, and increased levels of inflammatory cytokines.

### THBS1 amplifies inflammatory responses via NF-κB activation

To elucidate the mechanism by which THBS1 regulates inflammation, we investigated its interaction with the NF-κB pathway in LPS-stimulated hPDLFs (Figure S2). THBS1 overexpression significantly increased the phosphorylation ratio of p65 (p-p65/total p65) (Figure S2A) and elevated the secretion of IL-6 (Figure S2B), IL-1β (Figure S2C), and TNF-α (Figure S2D) compared to LPS-treated controls. Crucially, these effects were abrogated upon treatment with the NF-κB inhibitor BAY11-7082, which led to a reduction in the p-p65/total p65 ratio and diminished cytokine levels; in contrast, the DMSO solvent control group showed no significant alterations. These results demonstrate that THBS1 amplifies inflammatory responses by activating the NF-κB signaling pathway.

## Discussion

GCF offers distinct advantages over saliva for studying molecular changes in the local microenvironment in periodontitis. This is because GCF originates directly from the periodontal tissue spaces. Its composition includes various bioactive molecules, including cytokines, enzymes, and non-coding RNAs (ncRNAs) [16, 17]. Among these, miRNAs have become a research hotspot for non-invasive diagnosis of periodontitis due to their high stability and ease of detection. For example, studies have shown that miR-1226 expression in GCF correlates significantly with periodontitis severity, with ROC curve validation supporting its role as a potential diagnostic marker (AUC = 0.866) [18]. Furthermore, miR-221-3p is notably under expressed in the GCF of patients with periodontitis, indicating its potential utility in the diagnosis and assessment of the condition (AUC = 0.880) [19]. Our study reveals significantly lower miR-671-5p expression levels in the GCF of periodontitis patients compared to healthy controls, achieving an AUC of 0.887, comparable to established miRNA biomarkers. This miRNA demonstrates excellent diagnostic performance, achieving an AUC of 0.887. Further analysis indicated that patients exhibiting reduced miR-671-5p expression presented significantly higher proportions of deep periodontal pockets, greater clinical attachment loss, and a higher frequency of bleeding on probing than those with elevated expression. Additionally, miR-671-5p levels correlated significantly with pro-inflammatory cytokine levels. These clinical parameters directly reflect periodontitis severity and are closely linked to tissue destruction and inflammatory amplification [20, 21]. This suggests that miR-671-5p may not only serve as a potential marker for the diagnosis of periodontitis but may also play a role in the progression of periodontitis.

hPDLFs are critical for the homeostasis and repair of periodontal tissue. In the context of periodontitis, chronic inflammation significantly impairs the functions of hPDLFs, leading to reduced viability, diminished migratory capabilities, and excessive secretion of pro-inflammatory cytokine, which collectively exacerbate tissue destruction [22]. To quantitatively assess these functional impairments, we employed CCK-8 and Transwell assays-established methodologies recognized for their precision in quantifying proliferative capacity and directional cell movement in inflammation studies [23], aligning with standardized approaches for evaluating cellular responses to pathological stimuli. Understanding molecular mechanisms regulating PDLFs is essential for unraveling periodontitis pathology and developing therapies. Previous studies show specific miRNAs regulate hPDLFs responses to stimuli like LPS: miR-146a suppresses pro-inflammatory cytokines via TRAF6/p38 MAPK [24], and miR-29a modulates cell function through the Wnt pathway [25]. This study represents the first to investigate the role of miR-671-5p in regulating hPDLFs function in periodontitis. In vitro, miR-671-5p expression decreased dose-dependently under LPS-simulated inflammatory conditions. Notably, the overexpression of miR-671-5p significantly enhances both the viability and migration capacity of hPDLFs exposed to LPS, concurrently attenuating the inflammatory response. This finding is consistent with the understood role of miR-671-5p in various inflammation and injury models across different tissues. For instance, miR-671-5p downregulation is also associated with decreased cell viability and increased inflammation in myocardial ischemia/reperfusion injury [26]. In osteoarthritis, targeted inhibition of miR-671-5p promotes disease progression [27]. In chondrocytes, the lncRNA DLEU1 affects inflammation by regulating miR-671-5p [28]. These findings suggest that miR-671-5p likely exerts protective effects on the viability and migratory capabilities of hPDLFs while also modulating inflammation. Thus, it offers a novel molecular perspective for understanding cellular dysfunction observed in periodontitis. However, it is important to note that this study utilized a singular LPS stimulus model. Future studies should include clinically relevant pathogens like *Porphyromonas gingivalis* to validate the role of miR-671-5p in complex infectious microenvironments.

Using a dual-luciferase reporter assay, we further confirmed that miR-671-5p directly targets the 3’UTR of THBS1 mRNA. THBS1 is a multifunctional matricellular protein. In a skeletal muscle atrophy model, THBS1 promotes autophagy and proteasomal degradation pathways by inducing ATF4 expression, leading to muscle mass loss [29]. In pulmonary fibrosis, miR-335-3p targets THBS1; overexpression of THBS1 exacerbates TGF-β1-induced epithelial-mesenchymal transition [30]. Notably, a recent periodontitis study found that circLRRC4C upregulates THBS1 expression by sponging miR-485-3p. This upregulation promotes apoptosis and inflammation in PDLFs. Inhibiting THBS1 significantly alleviated periodontal tissue destruction in mice [31]. Our findings align coherently with these prior studies, as THBS1 overexpression counteracted the protective effects elicited of miR-671-5p mimics on PDLFs. This evidence positions THBS1 as a core downstream target of miR-671-5p. Furthermore, we demonstrate that THBS1 activates NF-κB signaling—increasing p65 phosphorylation and cytokine production—and this effect is abolished by NF-κB inhibition (Figure S2). This mechanistically resolves that THBS1 influences IL-6, IL-1β, and TNF-α expression indirectly through NF-κB activation, rather than direct transcriptional regulation. This NF-κB-dependent mechanism aligns with recent findings in pneumonia models [32]. Hence, the protective effects of miR-671-5p are mediated by disrupting this THBS1-NF-κB amplification cascade.

While our findings establish miR-671-5p as a promising diagnostic biomarker and anti-inflammatory mediator via THBS1 targeting, several limitations merit consideration. Firstly, the clinical translation of miR-671-5p mimics necessitates overcoming challenges with oral delivery, particularly enzymatic degradation within the gingival crevice and the need for targeted cellular uptake in periodontal tissues. Future investigations should reaffirm therapeutic efficacy in vivo utilizing established periodontitis models, such as P. gingivalis-infected rodents, and explore combinatorial strategies that integrate miRNA therapy with mechanical debridement or antibiotics to enhance regenerative outcomes. Secondly, although miR-671-5p demonstrates high diagnostic accuracy, its specificity as a periodontitis-exclusive biomarker requires further scrutiny. Given its dysregulation in arthritis, cardiovascular diseases, and other inflammatory conditions, comprehensive cross-disease comparisons of miR-671-5p expression profiles are essential to confirm clinical specificity. Thirdly, systematic network analysis integrating miRNA-mRNA interactions—as exemplified by Pan-cancer genetic analysis of cuproptosis and copper metabolism-related gene set [33], and pan-cancer studies dissecting cuproptosis gene sets or mitotic checkpoint kinases [34]—was not employed here. Applying such approaches could unravel broader regulatory networks governing miR-671-5p/THBS1 axis functions in pathogenesis of periodontitis. Lastly, while multi-miRNA panels, such as those combining miR-671-5p with miR-1226 and miR-221-3p, may offer enhanced diagnostic precision, the prolonged storage of our archived GCF samples (exceeding three years) has impeded the possibility of additional miRNA quantification. Future prospective cohorts will focus on validating combinatorial signatures to optimize clinical applicability.

## Conclusions

Overall, this study is the first to confirm that low expression of GCF miR-671-5p is significantly associated with periodontitis severity, possessing both diagnostic efficacy and pathological regulatory functions. In vitro mechanistic studies show that miR-671-5p inhibits hPDLFs inflammatory responses and promotes cell viability and migration by targeting THBS1, providing new insights into the pathogenesis of periodontitis. Despite certain limitations, the results suggest that miR-671-5p may emerge as a promising biomarker for non-invasive diagnosis of periodontitis and represent a viable target for gene therapy. Future research endeavors should undertake large-scale, multi-center studies to further validate its clinical significance, alongside utilizing in vivo models to comprehensively investigate the regulatory network of the miRNA-THBS1-signaling pathway. Such efforts will lay the groundwork for the development of miRNA-based therapeutic strategies aimed at combating periodontitis.

## Supplementary Information

Below is the link to the electronic supplementary material.

Supplementary Material 1: miR-671-5p inhibitor upregulates THBS1 expression in unstimulated hPDLFs.

Supplementary Material 2: THBS1 activates NF-κB signaling to amplify inflammatory responses in LPS-stimulated hPDLFs. (A) Quantification of p-p65 and total p65 protein levels. The p-p65/total p65 ratio was normalized to the control group. (B-D) Secretion of (B) IL-6, (C) IL-1β, and (D) TNF-α in cell supernatants measured by ELISA.

Supplementary Material 3

## Data Availability

The datasets used and/or analysed during the current study are available from the corresponding author on reasonable request.

## Abbreviations

AUC: Area under the curve
BOP: Bleeding on probing
CAL: Clinical attachment loss
DDK1: Dicckopf-1
GCF: Gingival crevicular fluid
hPDLFs: Human periodontal ligament fibroblasts
IL-1β: Interleukin-1β
IL-6: Interleukin-6
LPS: Lipopolysaccharide
miRNAs: MicroRNAs
MMPs: Matrix metalloproteinases
MT: Mutant
NC: Negative control
ncRNAs: Non-coding RNAs
NF-κB: Nuclear factor kappa B
OE: Overexpression
PD: Probing depth
qRT-PCR: Quantitative real-time PCR
ROC: Receiver operating characteristic
TNF-α: Tumor necrosis factor-alpha
WT: Wild-type
3’UTR: 3’ untranslated region
