# Supplementary figures and images for: miR-671-5p as a diagnostic biomarker and therapeutic target in periodontitis via THBS1 regulation
Source: Hereditas. 2025 Sep 25;162:185. doi: 10.1186/s41065-025-00546-w (PMC12465853; doi:10.1186/s41065-025-00546-w)

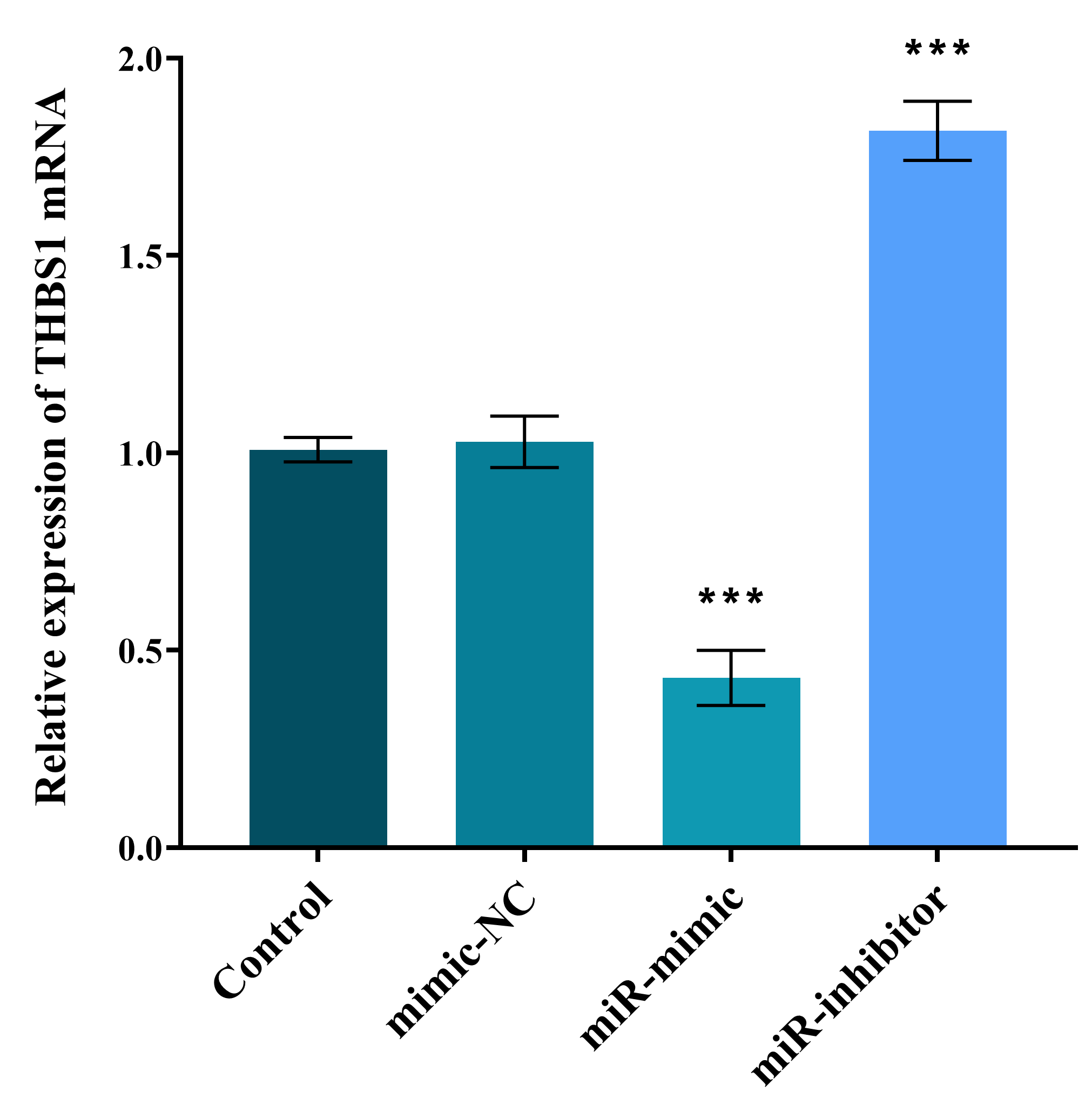

Supplement: Supplementary file 1 — Supplementary Material 1: miR-671-5p inhibitor upregulates THBS1 expression in unstimulated hPDLFs. [file 41065_2025_546_MOESM1_ESM.tif]

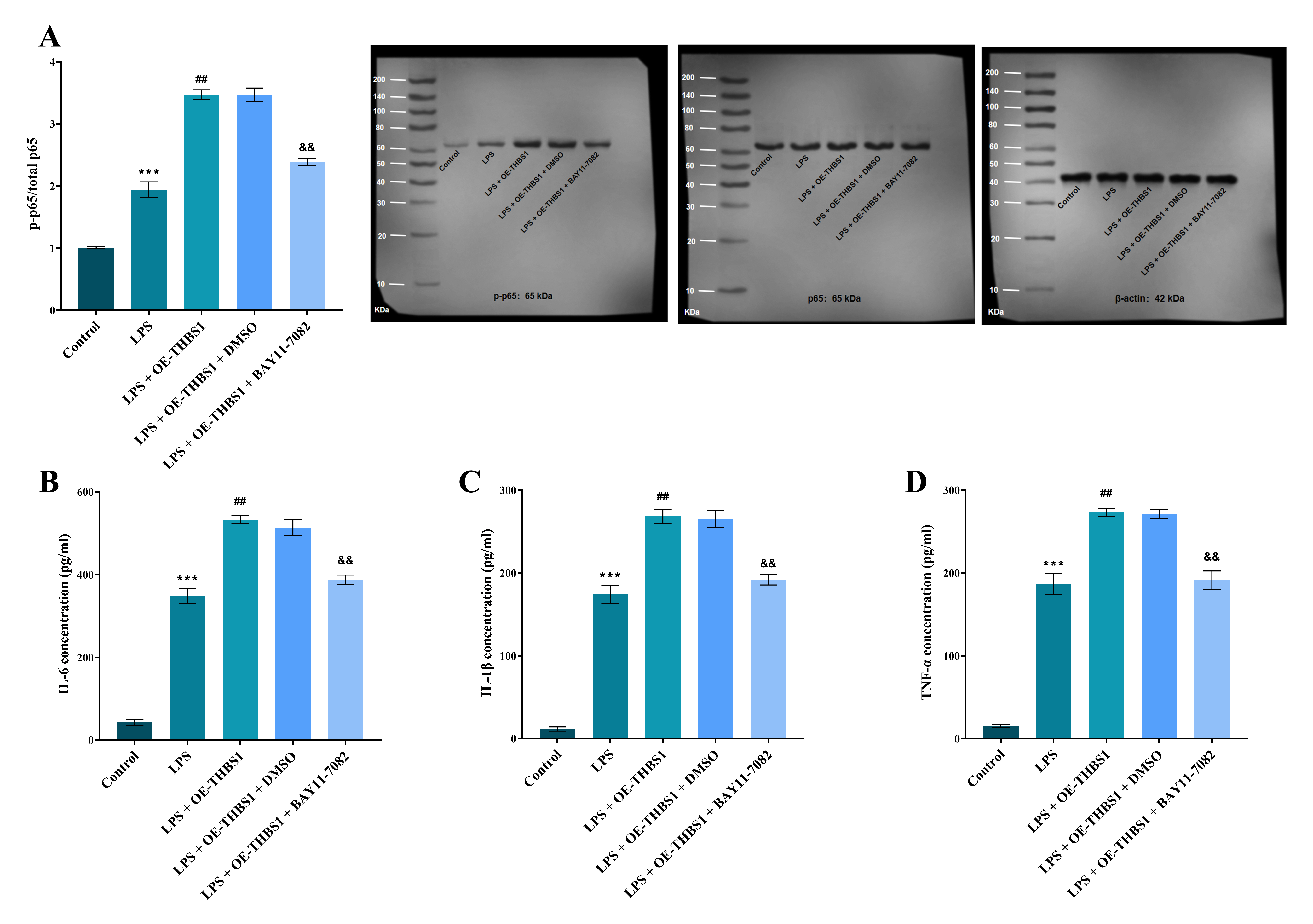

Supplement: Supplementary file 2 — Supplementary Material 2: THBS1 activates NF-κB signaling to amplify inflammatory responses in LPS-stimulated hPDLFs. (A) Quantification of p-p65 and total p65 protein levels. The p-p65/total p65 ratio was normalized to the control group. (B-D) Secretion of (B) IL-6, (C) IL-1β, and (D) TNF-α in cell supernatants measured by ELISA. [file 41065_2025_546_MOESM2_ESM.tif]
